# Advances in rehabilitation for bone and joint pain: a critical narrative review of evidence, gaps, and future directions

**DOI:** 10.3389/fpain.2026.1858635

**Published:** 2026-07-30

**Authors:** Jie Zhuang, Jinyan Wang, Yinhu Hu, Juan Jin, Jing Shi, Lei Song, Zekai Hu

**Affiliations:** 1 Department of Rehabilitation Medicine, The Second Rehabilitation Hospital Affiliated to Shanghai University of Traditional Chinese Medicine, Shanghai, China; 2 Baoshan Branch, Renji Hospital, School of Medicine, Shanghai Jiao Tong University, Shanghai, China; 3 School of Medicine, Shanghai University, Shanghai, China

**Keywords:** bone and joint pain, exercise therapy, manual therapy, narrative review, rehabilitation

## Abstract

Chronic bone and joint pain is highly prevalent, and rehabilitation is a core component of care. This critical narrative review uses a transparent, structured evidence-mapping approach to examine rehabilitation studies published from January 2021 to April 2026, integrate findings across intervention classes, and identify evidence gaps. Across the included evidence base, exercise therapy supports pain and functional outcomes in selected musculoskeletal conditions, although optimal dose and phenotype-specific matching remain uncertain. Manual therapy and physical modalities may provide short-term adjunctive symptom relief, but their effects are conditional on technique, context, and protocol. Digital rehabilitation and virtual-reality-based approaches can improve access and engagement, but functional and long-term benefits are heterogeneous. Low-load blood-flow-restriction training is promising where high external loads are not tolerated; safety, dose, and longer-term effectiveness require further study. Rather than implying a universal precision-rehabilitation model, we present phenotype-informed treatment matching as a research agenda. Key limitations include heterogeneity of populations, interventions, outcome measures, and follow-up, the selective nature of narrative synthesis, publication bias, and the absence of pooled quantitative estimates. Exercise remains a foundation of care, with adjuncts selected according to presentation, patient priorities, feasibility, and response to reassessment.

## 1 Introduction

Chronic bone and joint pain affects over 1.7 billion people globally, with osteoarthritis (OA) alone contributing to more than 170 million years lived with disability (1). Despite decades of research, a troubling paradox remains: up to 20%–30% of patients undergoing total knee arthroplasty report persistent pain and dissatisfaction (2–4), and many patients with chronic low back pain continue to suffer despite guideline-concordant care (5, 6). This gap between evidence and outcome suggests that our current conceptual frameworks may be incomplete. Moreover, the economic burden is staggering: lost productivity, healthcare utilization, and disability payments related to joint pain cost developed economies billions annually. Yet, the proportion of research funding allocated to non-pharmacological, non-surgical rehabilitation remains disproportionately small compared to pharmaceutical and device development.

The history of joint pain rehabilitation reveals a progression through three overlapping paradigms. The biomechanical paradigm (dominant 1960s–1990s) focused on structural pathology—cartilage degradation, ligament insufficiency, muscle weakness—and assumed that correcting these deficits would relieve pain. This paradigm gave us exercise therapy, manual therapy, and surgical reconstruction, but it struggled to explain why many patients with severe radiographic OA are asymptomatic and why others with minimal pathology experience debilitating pain (7). This discordance between structural pathology and pain experience has led researchers to explore alternative drivers of pain, including central sensitization, neuroinflammation, and psychological factors. The paradigm's greatest limitation was its implicit assumption of linear causality: more pathology equals more pain. When this failed to hold, the field was left without a coherent explanation.

The biopsychosocial paradigm (emerging 1990s–present) expanded the focus to include psychological (catastrophizing, fear, self-efficacy), social (work status, support networks), and contextual factors. This shift, championed by Engel and later integrated into pain rehabilitation, has improved outcomes but remains incompletely implemented in routine care (8, 9). A critical observation is that the biopsychosocial model is often invoked but rarely operationalized. Many clinicians pay lip service to psychosocial factors while continuing to practice within a purely biomedical framework.

An emerging research direction is to use phenotypic characteristics (for example, sensorimotor, psychological, and neurophysiological features) to test whether treatment can be better matched to individual patients. Early prediction and classification studies illustrate this potential (10). Preoperative psychological and clinical data have also been evaluated as predictors of persistent pain after total knee arthroplasty (11). However, these approaches remain hypothesis-generating rather than established clinical decision rules. Their clinical value requires externally validated treatment-by-phenotype effects, feasible implementation, and demonstration that matching improves outcomes beyond usual care.

This critical narrative review was designed to map and interpret a broad, heterogeneous rehabilitation literature rather than to estimate a single pooled effect. Its aims were to: (1) synthesize cross-cutting findings across intervention domains; (2) identify consistent findings, contradictions, and evidence gaps; (3) distinguish foundational treatments from adjunctive and emerging options; and (4) propose a research agenda. We focus on common conditions (knee/hip osteoarthritis, patellofemoral pain, low back pain, shoulder disorders, temporomandibular disorders, and post-arthroplasty pain) and interventions (exercise, manual therapy, physical modalities, digital health, interventional procedures, and psychological approaches).

## 2 Methods

### 2.1 Scope, sources, and search strategy

We searched PubMed, Embase, Web of Science Core Collection, and PEDro for records published from 1 January 2021 to 30 April 2026. The complete database-specific search strings, fields, filters, and the number of records identified are reported in Supplementary Table 3. Searches were designed to identify recent controlled trials, systematic reviews/meta-analyses, and clinically relevant guidance on bone and joint pain rehabilitation. The reported database yields and flow diagram are provided for transparency; their use does not alter the classification of this article as a critical narrative review.

### 2.2 Eligibility, selection, and data charting

We included randomized controlled trials, systematic reviews and meta-analyses, high-quality prospective cohort studies, and clinical practice guidelines that addressed rehabilitation interventions for bone and joint pain. We excluded case reports (except where they clarified rare conditions), animal studies, non-English publications without sufficient information for appraisal, retracted articles, conference-only reports, and duplicate or overlapping datasets. Two reviewers screened titles/abstracts and assessed full texts against these criteria; disagreements were resolved by discussion and consensus. Because this was a narrative review, we did not calculate an inter-reviewer agreement statistic and did not use a protocol-registered, exhaustive selection process. Instead, purposive evidence mapping prioritised methodological transparency, relevance to the review questions, coverage of intervention domains, and explanatory value for areas of agreement or uncertainty.

### 2.3 Evidence appraisal and narrative synthesis

PEDro and AMSTAR-2 assessments (Supplementary Table 1, 2) were used as structured contextual aids to interpreting individual reports (12) and systematic reviews (13); they were not used to generate a pooled certainty rating or a threshold-based inclusion rule. Two reviewers independently completed the appraisals, and disagreements were resolved by consensus. No inter-reviewer agreement metric was calculated.

To support reproducibility, we charted condition/population, intervention and comparator, design, sample, outcomes, follow-up, methodological limitations, and relevance to the review questions using a structured data-extraction form. Evidence was synthesized narratively by intervention domain, with explicit attention to convergence, inconsistency, clinical applicability, and knowledge gaps. We did not perform a *de novo* quantitative meta-analysis.

## 3 Results and evidence map

**Figure 1:**
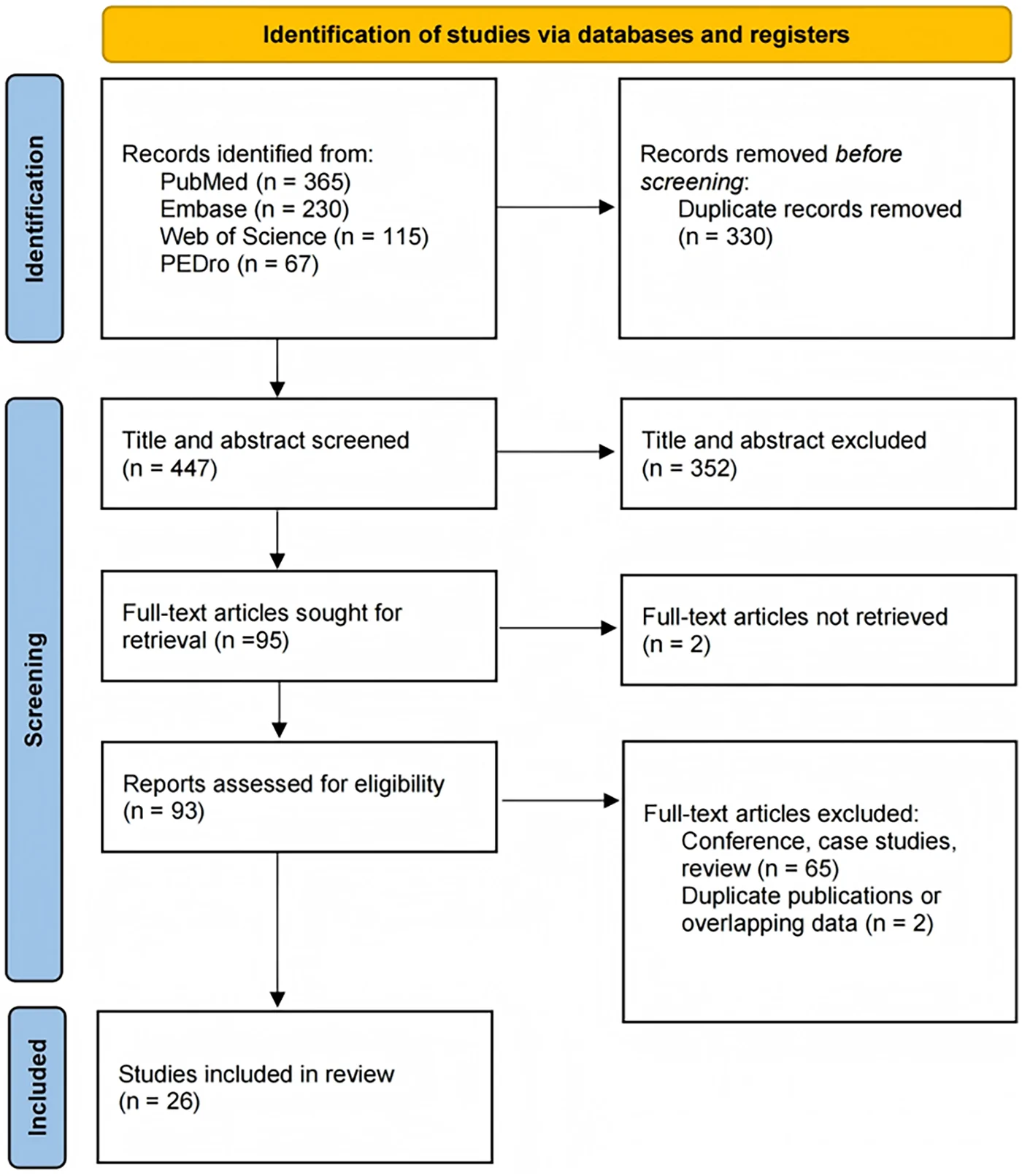
PRISMA 2020 flow diagram of study selection. A total of 777 records were identified from PubMed (*n* = 365), Embase (*n* = 230), Web of Science (*n* = 115), and PEDro (*n* = 67). After removal of duplicates (*n* = 330), 447 records were screened by title and abstract, and 352 were excluded. 95 full-text articles were sought for retrieval, of which 2 were not retrieved. 93 reports were assessed for eligibility. Exclusions included conference papers, case studies, and reviews (*n* = 65), as well as duplicate publications or overlapping data (*n* = 2). Finally, 26 studies were included in the review.

The database searches identified 777 records (PubMed, *n* = 365; Embase, *n* = 230; Web of Science, *n* = 115; PEDro, *n* = 67). After removing 330 duplicates, 447 titles/abstracts were screened and 93 full texts were assessed; 26 sources were selected to inform the narrative evidence map. Figure 1 documents these steps transparently; the article remains a critical narrative review. Basic characteristics of the mapped evidence are shown in Table 1.

**Table 1:** Basic characteristics of included studies.

First Author (Year)	Study Type	Condition/Population	Intervention of Interest	Comparator	Sample Size (*N*)	Main Outcomes Measured	Key Finding/Conclusion
META-ANALYSES & SYSTEMATIC REVIEWS							
Lin et al. (2025) (23)	Systematic Review & Meta-analysis	Knee Osteoarthritis (KOA)	Low-load Blood Flow Restriction (BFR) training	High-load resistance training or sham/control	10 RCTs	Pain, muscle strength, physical function	Low-load BFR training is effective for improving pain and muscle strength in KOA, and may be an alternative to high-load training.
Pamboris et al. (2024) (17)	Systematic Review & Meta-analysis	ACL reconstruction (ACLR) & ACL-deficient knees	Open Kinetic Chain (OKC) exercises	Closed Kinetic Chain (CKC) exercises	9 studies (*N* = 433)	Knee laxity, quadriceps strength, function, pain	OKC exercises are superior for improving quadriceps strength 3–4 months post-ACLR and as effective as CKC for knee laxity, supporting their inclusion in early rehabilitation.
Jalili Bafrouei et al. (2025) (25)	Systematic Review & Meta-analysis	ACL Reconstruction (ACLR)	Core Exercises (CE)	Traditional exercise	10 RCTs (*N* = 463)	Pain (VAS), Function (IKDC, Lysholm), ROM, hop tests	CE significantly improves functional performance (IKDC, hop tests) but has no significant effect on pain relief compared to traditional exercise.
Li et al. (2024) (31)	Meta-analysis	Older adults (≥60 years) with/without knee OA	Photobiomodulation therapy (PBMT) + Exercise	Exercise only or sham laser + exercise	14 RCTs	WOMAC (pain, stiffness, function), VAS, ROM, muscle strength	PBMT combined with exercise provides additional benefits for pain relief, knee joint function, and ROM, but not for muscle strength or mobility tests (TUG, 6MWT).
Luo et al. (2025) (37)	Network Meta-analysis	Musculoskeletal disorders (MSDs)	13 AI-assisted rehabilitation strategies (e.g., exergaming, robotics, telerehab)	Conventional or usual care	33 RCTs	Pain, functional outcomes, ROM	Therapeutic exergaming (pain), gamified exergaming (function), and single-joint rehab robots (ROM) were most effective. AI-assistance outperforms usual care.
Xiang et al. (2023) (38)	Systematic Review & Meta-analysis	Knee Osteoarthritis (KOA)	Telerehabilitation strategies	Conventional treatment or usual care	6 RCTs (*N* = 734)	Pain (WOMAC), physical function (WOMAC)	Telerehabilitation significantly improves pain but not physical function in KOA patients. No specific strategy was superior.
Xie et al. (2021) (39)	Systematic Review & Meta-analysis	Knee Osteoarthritis (KOA)	Internet-based rehabilitation programs	Conventional rehabilitation	6 RCTs (*N* = 791) for review; 4 RCTs (*N* = 411) for meta-analysis	Pain (WOMAC), physical function (WOMAC)	Internet-based rehabilitation significantly improves pain but not physical function compared to conventional rehabilitation.
Long et al. (2025) (40)	Systematic Review & Meta-analysis	Osteoarthritis (OA)	Digital exercise therapy (app, website, phone)	Conventional treatment	9 RCTs (*N* = 1,604)	Pain (NRS), physical function (WOMAC)	Digital exercise therapy significantly improves pain and physical function immediately post-intervention, but only physical function benefits are sustained at follow-up.
Nayab et al. (2024) (41)	Systematic Review & Meta-analysis	Hip & Knee Osteoarthritis	Various exercises (aerobic, resistance, Tai Chi, multimodal)	Standard care, alternative exercise, or placebo	12 studies (*N* = 4,920)	Pain, physical function, quality of life (QoL)	Exercise interventions are effective. Tai Chi/Baduanjin benefit older adults; aerobic training benefits younger individuals. Diet + exercise produced the largest effect.
Xie et al. (2026) (35)	Systematic Review & Meta-analysis	Total knee arthroplasty (TKA)/Total hip arthroplasty (THA)	Cryotherapy (various modalities)	Non-cryotherapy (e.g., no intervention, compression)	21 RCTs (*N* = 1,462)	Pain, analgesic consumption, blood loss, ROM, adverse events	Cryotherapy aids rehabilitation. Effects differ between TKA (reduces pain, blood loss) and THA (reduces pain only). No effect on ROM, swelling, or hospital stay.
RANDOMIZED CONTROLLED TRIALS (RCTs)							
Zhang et al. (2024) (14)	RCT	Older adults with sarcopenia	Low-load BFR training (LRT-BFR) (20%–30% 1RM)	Conventional high-intensity resistance training (CRT) (60%–70% 1RM)	21 (BFR=10, CRT=11)	Knee extensor strength (KES), appendicular skeletal muscle mass index (ASMI), physical performance, CVD risk factors, biomarkers	Both effective. CRT superior for muscle mass; LRT-BFR superior for cardiovascular health (HR). LRT-BFR is a potential alternative.
Massah et al. (2025) (16)	RCT	Chronic non-specific low back pain (CNLBP)	Single session of Virtual Reality Exergames (EXG)	Single session of Core Stability Exercises (CSEs)	40 (EXG=20, CSEs=20)	Pain (VAS), cognitive factors (attention, working memory), mood, fear-avoidance beliefs (FAB)	A single EXG session was more effective than CSEs in improving reaction time, pain, FAB, and positive mood states.
Werasirirat et al. (2026) (15)	RCT	Non-specific chronic low back pain (NSCLBP)	BFR training + Core Stabilization Exercise (CSE)	CSE alone	38 (BFR + CSE=19, CSE=19)	Muscle activity (EMG), muscle thickness (ultrasound), disability (MODQ)	BFR combined with CSE for 4 weeks is more effective than CSE alone in improving TrA, MF, and Gmax muscle activity, thickness, and disability.
Erayata & Menek (2025) (19)	RCT	Post-ACL reconstruction	Percussion massage therapy (PMT) + structured exercise program	Structured exercise program alone	24 (PMT=12, Exercise=12)	Pain (VAS), ROM, functionality (WOMAC), balance (Berg), QoL (SF-36)	Both groups improved. PMT group was superior in ROM, joint position sense, pain, functionality, and balance, suggesting PMT is an effective adjunct.
Menek & Dansuk (2025) (18)	RCT	Conservatively treated distal radius fracture (DRF)	Closed kinetic chain (CKC) exercises + conventional physiotherapy	Conventional physiotherapy alone	40 (CKC=20, Conventional=20)	Pain (VAS-activity), ROM, function (DASH), joint position sense (JPS)	Both groups improved. CKC group showed significantly greater improvements in all outcomes, especially proprioception and function.
Sremakaew et al. (2023) (20)	RCT	Chronic neck pain with sensorimotor impairments	Local neck treatment (NT) + JPS/oculomotor (OC) and/or balance (B) exercises	Local neck treatment (NT) alone	152 (4 groups, n≈38 each)	Joint position sense (JPS), postural sway, pain, disability (NDI), ROM, gait speed	Adding specific JPS/OC and balance training to NT best improved cervical proprioception and balance. Sensorimotor training improved long-term maintenance of pain/disability.
Karabay et al. (2025) (22)	RCT	Subacromial pain syndrome (SPS)	Isolated eccentric strengthening + multimodal physiotherapy program (MPP)	Isolated concentric strengthening + MPP or MPP alone	45 (ESG=15, CSG=15, CG = 15)	Function (Constant-Murley Score, DASH), pain (VAS), isometric/eccentric strength (HHD), joint position sense (JPS)	Eccentric training provided added benefits for abduction strength and JPS during abduction, but no clinically superior effects on function, pain, or external rotation strength compared to concentric training or MPP alone.
Pozsgai et al. (2022) (27)	RCT	Knee Osteoarthritis (KOA)	Single session of end-range Maitland mobilization	Single session of not end-range Maitland mobilization or sham	66 (end-range=21, not end-range=20, sham=22)	Pressure pain threshold (PPT—local & distant), Timed Up and Go (TUG), passive resistance	Single end-range Maitland mobilization was effective immediately and for up to 4 days on pain sensitization and physical function, superior to the other groups.
Ocalan et al. (2025) (28)	RCT	Cervical myofascial pain syndrome (MPS)	Myofascial release therapy (MRT) + standard physical therapy	Standard physical therapy alone	60 (MRT=30, Control=30)	Pain (VAS), trigger point (TP) number, PPT, ROM, disability (NDI), QoL (Nottingham Health Profile)	MRT added to standard therapy provided significantly better results in pain, TP numbers, PPT, ROM, disability, and QoL.
Bağcıer et al. (2023) (29)	RCT	Hemiplegic shoulder pain (HSP) post-stroke	Dry needling (DN) + conventional rehabilitation	Conventional rehabilitation alone	38 (DN = 19, Control=19)	Pain (VAS), ROM, functionality (QuickDASH, Fugl-Meyer Assessment Upper Extremity)	Both groups improved. Adding DN showed statistically superior short-term results for pain and ROM, but differences were not clinically significant (MCID) and not sustained at 3 months.
Zhang et al. (2023) (34)	RCT	Post-stroke hemiplegic shoulder pain (HSP)	Ultrasound-guided betamethasone local injection (UGLI) + rehabilitation	Extracorporeal shock wave therapy (ESWT) + rehabilitation	42 (UGLI=21, ESWT=21)	Pain (VAS), function (Neer, FMA), ROM, inflammatory cytokines	Both improved pain and function. UGLI had better short-term analgesia and reduced serum cytokines more than ESWT.
Neculăț et al. (2024) (33)	RCT (quasi-experimental)	Patellofemoral pain syndrome (PFPS)	Radial shockwave therapy (rESWT) + functional rehabilitation program	Traditional electrotherapy (TENS, ultrasound, laser) + functional rehab	64 (rESWT=32, Traditional=32)	Pain (VAS), knee flexion ROM, suprapatellar perimeter (muscle mass)	Both improved. Shockwave group showed significantly greater improvement in pain and ROM, suggesting additional benefits.
Lee et al. (2026) (24)	RCT	Patellofemoral pain syndrome (PFPS)	Low-intensity multi-joint BFR training	Same multi-joint exercise without BFR	41 (BFR=20, Control=19)	Pressure pain threshold (PPT), isometric muscle strength, muscle tone/stiffness (MyotonPRO), balance (Y-Balance Test)	BFR group showed significantly greater improvements in strength, pain sensitivity, balance, and muscle mechanical properties (tone, stiffness).
Quesnot et al. (2024) (36)	RCT	Post-total knee arthroplasty (TKA)	Compressive cryotherapy (Game Ready) + rehabilitation	Standard cryotherapy (ice packs) + rehabilitation	40 (Compressive=20, Standard=20)	ROM (flexion/extension), swelling (circumference), pain (VNRS), function (6MWT, KOOS)	Both improved. Compressive cryotherapy led to faster improvement in ROM and swelling, and better outcomes in activity pain, walking distance, and KOOS.
Tabassum et al. (2024) (26)	RCT	Chronic neck pain	Muscle Energy Technique (MET) or Facet Joint Mobilisation (FJM)	Conventional physical therapy (CPT)	105 (MET=35, FJM=35, CPT=35)	Pain (NPRS), disability (NDI), cervical lordosis (x-ray), ROM	MET and FJM were more effective than CPT. MET was superior for flexion/rotation; FJM was superior for cervical lordosis and extension.

### 3.1 Exercise therapy: foundations, evolution, and clinical translation

#### 3.1.1 Historical context and conceptual evolution

Exercise has been prescribed for joint pain since antiquity, but the mechanistic rationale has shifted dramatically. Early 20th-century approaches emphasized “joint sparing” (avoiding loaded movement). The 1970s–80s saw the rise of isokinetic strengthening, focusing on quadriceps deficits. The 1990s introduced closed kinetic chain principles from sports medicine. The 2000s brought neuromuscular and sensorimotor training. Most recently, low-load blood flow restriction (BFR) training challenges the high-load dogma (14).

Contemporary exercise programmes often combine strengthening, motor control, education, and other components. This makes it difficult to identify active ingredients, compare doses, and reproduce the elements responsible for benefit. The available literature therefore supports cautious interpretation of complex programmes and a need for replication and component-level evaluation, particularly for newer interventions such as blood-flow restriction and virtual-reality-based exercise (15, 16).

#### 3.1.2 Open vs. Closed Kinetic Chain: An Ongoing Debate

The Open Kinetic Chain/Closed Kinetic Chain (OKC/CKC) debate originated in Anterior Cruciate Ligament (ACL) rehabilitation, where concerns about anterior tibial translation during OKC knee extension led to CKC preference. A high-quality meta-analysis by Pamboris et al. found that OKC exercises were superior for quadriceps strength at 3–4 months post-Anterior Cruciate Ligament Reconstruction (ACLR) and equally effective for knee laxity (17). For ACL-deficient knees, low-load OKC showed no laxity difference vs. controls, while high-load OKC reduced laxity at 6 weeks (*p* = 0.02) but not 12 weeks.

Available comparisons do not establish a universal superiority of either open- or closed-kinetic-chain exercise. The certainty is limited by small samples, heterogeneity in loading parameters and outcomes, and incomplete blinding. Carefully dosed open-kinetic-chain exercise may be considered when clinically appropriate, but treatment selection should be based on the rehabilitation phase, tissue tolerance, functional goals, and patient response rather than kinetic-chain category alone.

A knowledge gap is that no studies have directly compared OKC vs. CKC in patellofemoral pain, where patellofemoral joint stress differs markedly between exercise types. This is a glaring omission given that Patellofemoral Pain Syndrome (PFPS) is one of the most common knee conditions. Interestingly, a recent randomized controlled trial in individuals with distal radius fractures found that a CKC exercise program was superior to conventional therapy for improving proprioception, upper extremity function, and pain, suggesting that the benefits of CKC training may extend beyond the knee joint (18).

#### 3.1.3 Proprioceptive and sensorimotor training: indications for effectiveness

Impaired joint position sense (JPS) is well-documented in chronic neck pain, after ACLR, and in knee OA (18, 19). A rigorous RCT by Sremakaew et al. found that adding sensorimotor training (JPS/oculomotor + balance exercises) to manual therapy and exercise produced sustained improvements in neck pain and disability at 6 and 12 months, with medium-to-large effect sizes (20).

However, the story is not uniform. For rotator cuff-related shoulder pain, a high-quality RCT by Buccioli et al. found no additional benefit of adding proprioceptive exercises to strengthening alone for Shoulder Pain and Disability Index (SPADI) scores (group × time interaction *p* = 0.25). Both groups improved significantly, but the addition was redundant (21).

Synthesis indicates that proprioceptive training is beneficial when JPS deficits are documented (neck, ACLR, OA) but may be unnecessary in conditions where proprioception is relatively preserved (some shoulder populations). The field needs standardized JPS assessment to target training appropriately. A historical note is that the assumption that “more is better” (adding proprioceptive training to everything) has likely wasted clinical resources. A pragmatic recommendation for clinicians is to screen for proprioceptive deficits using simple clinical tests—such as the joint position reproduction test with a goniometer—before adding sensorimotor components to an exercise program. If deficits are absent, additional proprioceptive training is unlikely to confer benefit beyond standard strengthening.

#### 3.1.4 Eccentric vs. Concentric Strengthening: Nuance Required

Eccentric exercise has been advocated for tendinopathies based on the “Alfredson protocol” (heavy slow eccentric loading). A well-designed RCT by Karabay et al. compared eccentric vs. concentric strengthening in subacromial pain syndrome (22). Both groups received multimodal physiotherapy. Eccentric training improved abduction strength and JPS more than concentric training, but pain relief did not differ. Synthesis suggests that the benefits of eccentric training may be specific to strength and sensorimotor outcomes, not analgesia. Clinicians should not expect superior pain relief from eccentric protocols. A broader implication is that this finding challenges the rationale for prescribing eccentric exercise primarily for pain relief. If pain reduction is the goal, concentric training may be equally effective and often easier for patients to perform. Moreover, eccentric exercises are associated with greater delayed-onset muscle soreness and may lead to poorer adherence in some populations, particularly older adults or those with high pain-related fear. Therefore, patient preference and tolerance should guide the choice between eccentric and concentric protocols when both are clinically appropriate.

#### 3.1.5 Low-Load blood flow restriction: A potential paradigm shift

BFR training represents a genuine conceptual advance, using low loads (20-30% 1RM) with vascular occlusion to produce strength gains comparable to high-load training. A meta-analysis by Lin et al. found that low-load BFR significantly improved knee pain (SMD=0.25), quadriceps strength (SMD=0.46), and 30-second sit-to-stand performance (WMD=1.71) in KOA compared to conventional resistance training (23).

Important limitations are that most studies had short follow-up (≤12 weeks). Occlusion pressure protocols varied widely (from 40% to 80% of limb occlusion pressure). Safety data are limited; although no serious adverse events were reported, transient muscle soreness and delayed-onset muscle soreness are common. Patients with venous thromboembolism risk were typically excluded, limiting generalizability. A conceptual concern is that the mechanisms of BFR remain debated. Is the effect primarily metabolic (hypoxia-induced muscle fiber recruitment), or is there a significant placebo component given the novel sensation of occlusion?

Low-load BFR is a potential option for selected patients who cannot tolerate conventional loading, under appropriate screening and supervision. The current evidence does not support routine first-line use or clinical superiority across populations. Longer follow-up, standardized occlusion protocols, systematic adverse-event reporting, and comparisons with well-dosed conventional exercise are needed. A recent patellofemoral-pain trial is consistent with short-term potential, but should not be extrapolated beyond its studied context (24).

#### 3.1.6 Core stabilization: From theory to evidence

Core stabilization exercises became wildly popular in the 2000s, driven by biomechanical theories linking deep abdominal and multifidus dysfunction to LBP. A meta-analysis by Bafrouei et al. on ACLR patients found that core exercises improved functional performance but had inconsistent effects on pain (25).

**Figure 2:**
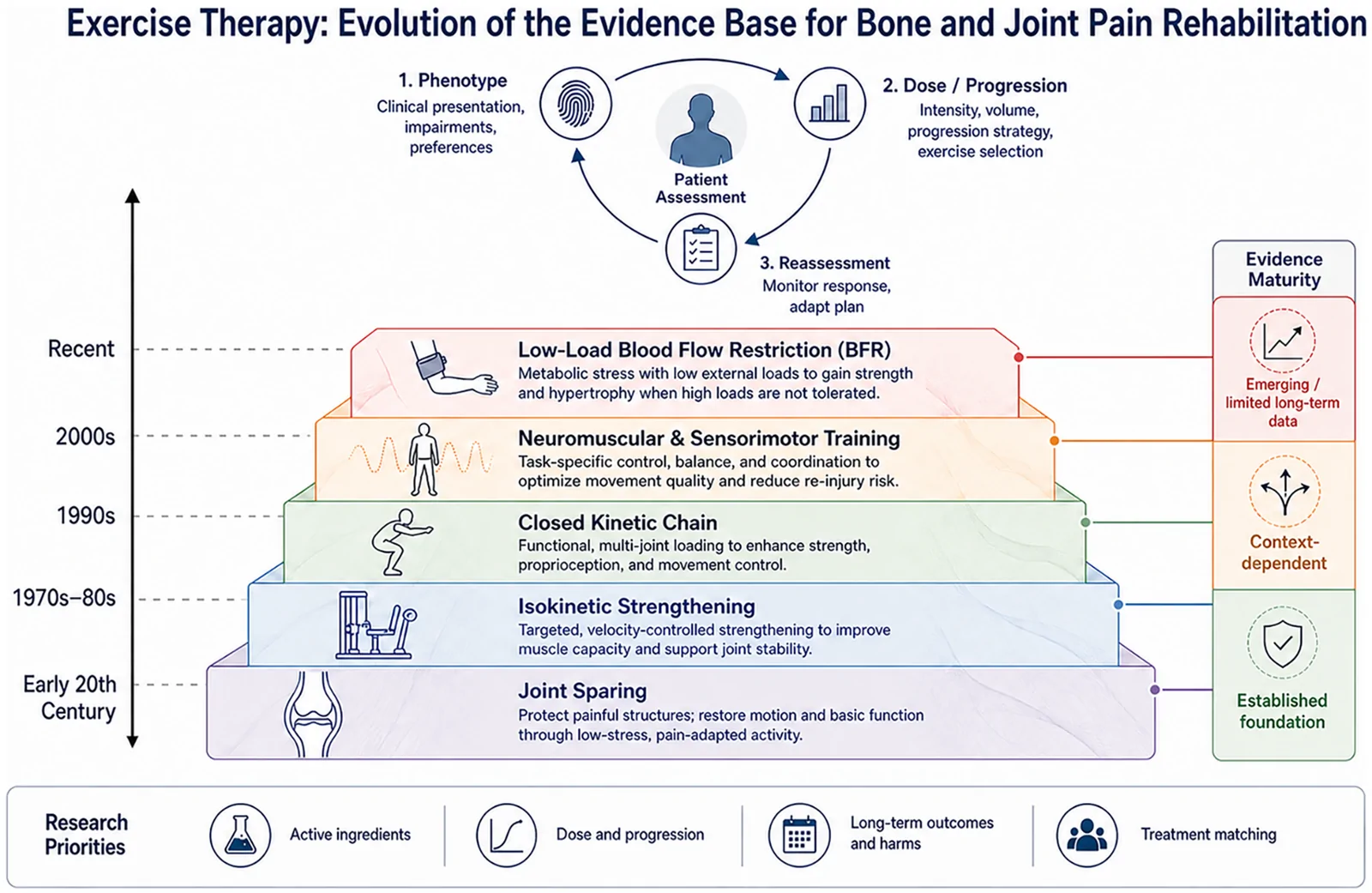
Exercise therapy: evidence-calibrated evolution and clinical translation. The layered timeline shows how major approaches were added to practice over time. The evidence-maturity band distinguishes established foundations from context-dependent and emerging strategies. The upper feedback loop links phenotype assessment, dose/progression, and reassessment; the lower panel identifies active ingredients, dose-response, long-term outcomes, and treatment matching as priorities. The figure is a conceptual narrative synthesis and does not imply comparative effect estimates.

Core stabilisation should not be assumed to provide benefit beyond general exercise for all patients with low back pain. It may be appropriate for people with identifiable movement-control impairments, but the evidence does not justify universal prescription. Assessment of movement, tolerance, goals, and preferences should guide whether targeted trunk exercises are added to a broader progressive programme. Figure 2 summarises this evidence-calibrated approach and the key questions that remain unresolved.

### 3.2 Manual therapy: mechanisms, specificity, and clinical utility

#### 3.2.1 The problem of specificity

Manual therapy has been practiced for centuries, but its specific mechanisms remain controversial. Does joint mobilization produce unique physiological effects beyond those of placebo touch? A RCT compared Muscle Energy Technique (MET), facet mobilization, and conventional physiotherapy for chronic neck pain (26). Both manual therapy groups were superior to conventional physiotherapy for pain and disability, but MET and mobilization did not differ from each other.

One interpretation is that the “specific technique” may matter less than the “specific context”—therapeutic alliance, expectation, and attention. This challenges the notion of technique superiority and suggests that manual therapy's benefits derive partly from non-specific factors. A research implication is that future trials should include sham manual therapy (credible placebo touch) to isolate specific effects. Few studies have done this. Moreover, the clinical implication is not that manual therapy is ineffective, but that the choice between different manual techniques may be less important than the quality of patient-clinician interaction and the delivery of a coherent treatment rationale. Clinicians should prioritize establishing a strong therapeutic alliance and providing clear expectations for recovery, rather than debating which specific mobilization technique is superior.

#### 3.2.2 Mobilization for osteoarthritis: end-range matters

A randomized trial compared end-range Maitland mobilization, non-end-range mobilization, and sham manual therapy for knee osteoarthritis (27). The reported short-term changes in pressure-pain threshold and function suggest that technique parameters may matter. However, the single-session design, knee-osteoarthritis population, and short follow-up do not establish a general rule for manual therapy. The findings should be interpreted as a rationale for testing patient- and protocol-specific parameters rather than as evidence of universal superiority of end-range mobilization.

#### 3.2.3 Myofascial release, Dry needling, and cupping: comparative effectiveness as adjuncts or alternatives

A RCT by Öcalan et al. found that myofascial release therapy added to standard physiotherapy for cervical myofascial pain syndrome produced superior outcomes in pain, trigger point numbers, Pressure Pain Threshold (PPT), Range of Motion (ROM), and Neck Disability Index (NDI) (28). For hemiplegic shoulder pain, dry needling improved range of motion but did not reach Minimal Clinically Important Difference (MCID) for pain (29). For Sacroiliac Joint (SIJ) dysfunction, dry cupping plus core stabilization was superior to core alone (30).

The overall picture suggests that these techniques are probably effective as adjuncts but unlikely to replace active exercise. The effect sizes are moderate, and long-term follow-up is lacking. One cautionary point is that the popularity of dry needling has outpaced its evidence base. Many of the included studies have high risk of bias (lack of sham control, inadequate blinding). Clinicians considering these modalities should first ensure that patients have completed an adequate trial of active exercise therapy (typically 4–6 weeks of supervised, progressive strengthening). If significant residual symptoms remain, adjunctive manual techniques may be offered as a second-line option, with clear discussion of the limited long-term evidence and the potential for placebo-driven improvements.

### 3.3 Physical modalities: adjuncts with modest effects

#### 3.3.1 Laser therapy: wavelength matters

Meta-analyses have reported that photobiomodulation added to exercise may improve selected pain and self-reported functional outcomes in knee osteoarthritis and musculoskeletal disorders (31, 32). The magnitude and clinical importance of these effects remain uncertain because protocols, comparators, and outcomes differ. Laser therapy should therefore be considered, if used, as a time-limited adjunct to active rehabilitation rather than a substitute for exercise. Independent replication and transparent reporting of device parameters and conflicts of interest remain important.

#### 3.3.2 Shockwave therapy: promising but protocol-dependent

A trial in patellofemoral pain reported greater short-term improvement with radial shockwave therapy plus rehabilitation than with a distinct electrotherapy programme (33). In hemiplegic shoulder pain, a comparison with corticosteroid injection suggested different time courses of analgesia (34). These findings are condition- and protocol-specific; they do not establish shockwave therapy as a broadly superior treatment. Standardized reporting of energy flux density, session number, comparator care, clinically important change, and cost-effectiveness is needed.

The available evidence indicates that shockwave is a reasonable option for refractory tendinopathy and some OA populations, but protocol standardization is urgently needed (energy flux density, frequency, number of sessions). One practical consideration is that the high cost of shockwave equipment limits access in many settings. Cost-effectiveness analyses are needed. Additionally, patients should be informed that shockwave therapy is often uncomfortable during administration and may require multiple sessions (typically 3–5) over several weeks to achieve maximal benefit. For patients who cannot afford or access shockwave, high-load eccentric exercise remains a well-established, low-cost alternative for tendinopathies.

#### 3.3.3 Cryotherapy: helpful for pain, not ROM

A meta-analysis by Xie et al. found that cryotherapy after TKA reduced pain on day 2 and reduced analgesic consumption, but did not improve ROM, patient satisfaction, or hospital stay (35). Compressive cryotherapy was superior to standard cryotherapy (36). A key takeaway is that cryotherapy is useful for acute pain control but should not be expected to improve functional outcomes. The very low to moderate evidence quality indicates that larger, better-controlled trials are needed. A clinical reality is that despite weak evidence, cryotherapy remains ubiquitous in postoperative protocols. This may reflect patient preference and perceived comfort rather than demonstrable efficacy.

### 3.4 Emerging technologies: current evidence and clinical potential

#### 3.4.1 Virtual reality: mixed evidence

A network meta-analysis by Luo et al. ranked therapeutic exergaming as most effective for pain relief (SUCRA=87.6%) and gamified exergaming for functional outcomes (SUCRA=99.6%) (37). However, a RCT by Cetin et al. found that adding Virtual Reality (VR) to traditional neck exercises provided no additional benefit for JPS, pain, or function. VR may be more effective for acute pain (distraction) than for chronic pain (where central mechanisms dominate). The specific population and VR protocol critically determine outcomes. A technology-agnostic point is that the novelty of VR may produce short-term effects that diminish with repeated use. Long-term studies are needed.

#### 3.4.2 Telerehabilitation: effective but not equivalent

Meta-analyses consistently show that telerehabilitation improves pain but not physical function compared to in-person care (38, 39). A more recent meta-analysis by Long et al. found that digital exercise therapy improved both pain (MD = −1.07) and function (MD = −2.39) immediately post-intervention, but pain relief was not sustained at follow-up (40).

**Figure 3:**
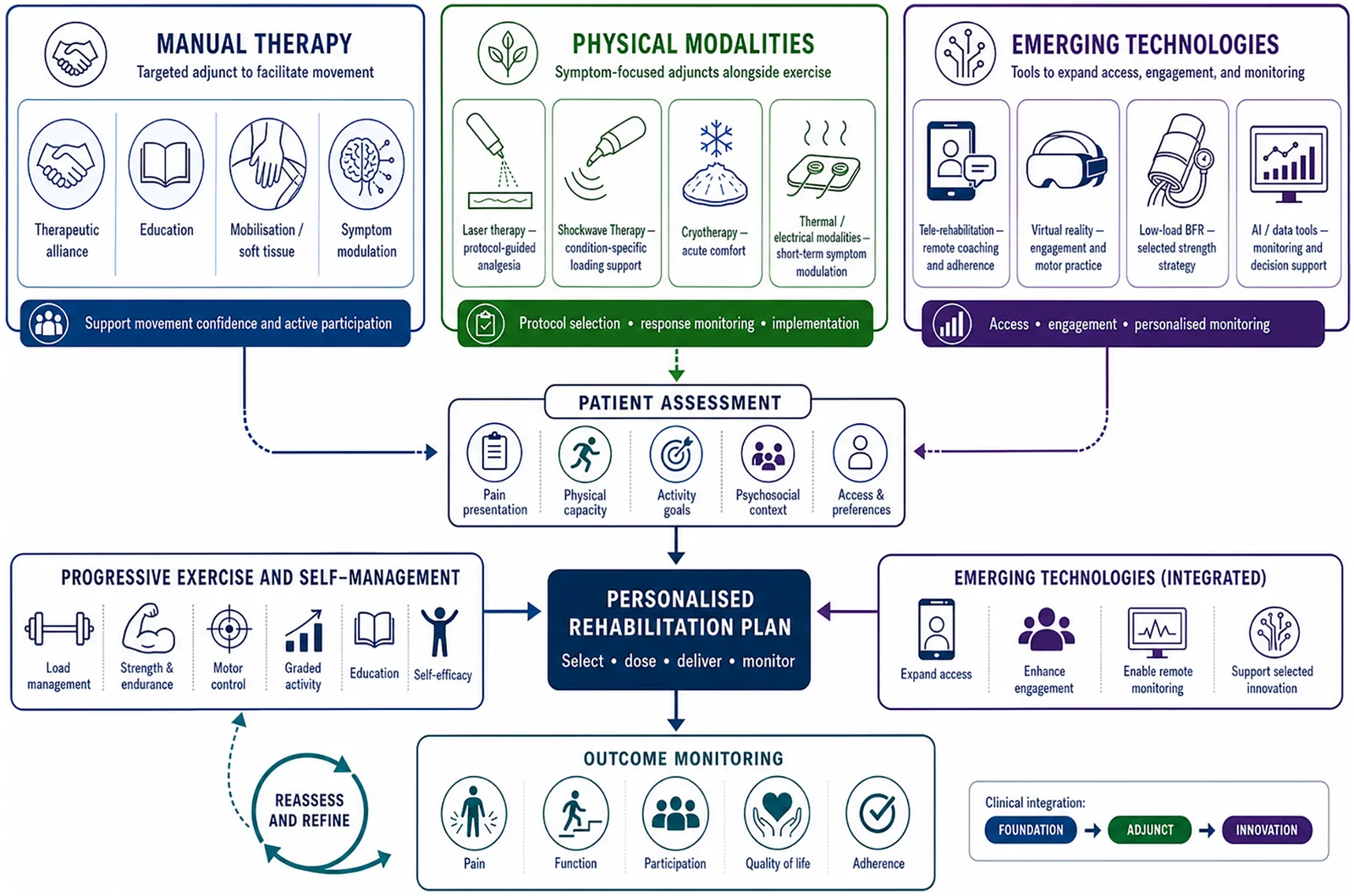
Integrated clinical pathway for rehabilitation strategies for bone and joint pain. Progressive exercise and self-management form the foundation of care. Manual therapy and physical modalities are integrated as targeted adjuncts, whereas tele-rehabilitation, virtual reality, low-load BFR, and data-enabled tools support access, engagement, monitoring, and personalised delivery. Patient assessment informs an individualised rehabilitation plan; outcome monitoring guides reassessment and refinement. The schematic summarises clinical integration and does not imply comparative effect estimates.

The reviewed evidence suggests that telerehabilitation can support access to exercise instruction and monitoring, particularly where face-to-face care is difficult to obtain. However, the available studies do not establish equivalence to in-person care, and functional and longer-term outcomes are variable. Hybrid models may be appropriate when initial assessment, movement observation, or complex barriers to participation require direct contact. Figure 3 places telerehabilitation and other technologies within an evidence-calibrated pathway rather than treating them as interchangeable replacements for exercise-based care.

### 3.5 Cross-cutting synthesis: evidence-calibrated clinical roles

Read across intervention categories, the literature supports three distinct clinical roles. Progressive exercise and self-management form the foundation of care; manual therapy and physical modalities are potentially useful adjuncts for selected, time-limited goals; and technologies such as telerehabilitation, virtual reality, BFR, and data-enabled tools remain context-dependent options with important implementation and long-term evidence gaps. This framing is intended to prevent both the overextension of promising modalities and the reduction of rehabilitation to a list of isolated techniques.

The central decision is therefore not whether one intervention is universally superior, but whether a treatment has a plausible role for a specific presentation, goal, preference, and setting, and whether benefit is confirmed on reassessment. Figures 2, 3 translate this synthesis into visual frameworks that explicitly display evidence maturity, conditional use, and research priorities.

## 4 Discussion

Across the mapped systematic reviews and trials, the direction and certainty of evidence vary by condition, intervention, comparator, and outcome. Exercise therapy has the most consistent role as a foundation of care in common musculoskeletal pain conditions, but the size and durability of benefit, optimal dose, and response modifiers remain uncertain (41). Manual therapy may provide short-term symptom relief in selected contexts, while the contribution of technique-specific vs. contextual effects remains unresolved (26, 42, 43). These findings support a calibrated, patient-centred interpretation rather than uniform recommendations across diagnoses.

Important inconsistencies remain. Effects of proprioceptive training differ between populations and may depend on baseline impairment (20, 21, 44). Effects of virtual reality are variable across acute and chronic pain contexts (37, 45), and core-stabilisation programmes have not shown consistent superiority over general exercise (25, 46). Heterogeneity in intervention content, co-interventions, comparators, outcome measures, and follow-up limits cross-study inference.

Persistent knowledge gaps limit progress. Dose-response data (optimal frequency, intensity, duration) are lacking for most conditions. Validated stratified care models do not exist: we cannot reliably predict which patients need exercise alone, added psychological support, or interventional procedures. Long-term follow-up (≥12 months) is rare, so whether short-term improvements alter the long-term trajectory of joint pain remains unknown. Cost-effectiveness data are sparse, particularly for emerging technologies like virtual reality and AI-assisted systems. Finally, implementation science is urgently needed; effective interventions are useless if not adopted in real-world settings with limited resources, variable clinician training, and diverse populations.

For researchers, priorities include better reporting of intervention components and dose, credible comparators or sham controls where appropriate, assessment of clinically important change and adverse events, longer follow-up, and prospectively tested treatment-matching hypotheses. For clinicians, exercise and self-management remain the starting point, while adjunctive modalities should be selected cautiously according to patient priorities, safety, feasibility, and observed response. For policymakers, implementation and equity questions—especially for telerehabilitation and technology-assisted care—require evaluation alongside effectiveness.

This review has important limitations. Although we used transparent electronic searches, dual screening, structured data charting, and contextual quality appraisal, it remains a critical narrative review rather than a protocol-registered systematic review. The search may not have captured all eligible evidence, and purposive selection and interpretation can introduce author bias. Marked heterogeneity in populations, interventions, comparators, outcomes, and follow-up precluded *de novo* quantitative synthesis. Publication bias, selective citation, and the under-representation of negative or null findings may further influence the conclusions. The revised text and figures therefore use calibrated language and explicitly identify uncertainty rather than assigning universal recommendations.

## 5 Conclusions

Exercise therapy and self-management remain the most consistently supported foundation for rehabilitation of bone and joint pain, but they should be individualized and progressed according to presentation, goals, tolerance, and response. Manual therapy and physical modalities may offer short-term adjunctive symptom relief for selected patients; emerging modalities such as low-load BFR, virtual reality, and technology-assisted rehabilitation are promising but require cautious, context-specific use.

Future studies should identify active ingredients, define dose-response relations, test phenotype-informed treatment matching, report safety and clinically important change, and establish long-term effectiveness and cost-effectiveness. A transparent narrative synthesis cannot resolve these uncertainties, but it can clarify the evidence boundaries and research priorities needed to move beyond one-size-fits-all rehabilitation.
