# Correction: Advances in rehabilitation for bone and joint pain: a critical narrative review of evidence, gaps, and future directions

**DOI:** 10.3389/fpain.2026.1953832

**Published:** 2026-08-26

**Authors:** Jie Zhuang, Jinyan Wang, Yinhu Hu, Juan Jin, Jing Shi, Lei Song, Zekai Hu

**Affiliations:** 1 Department of Rehabilitation Medicine, The Second Rehabilitation Hospital Affiliated to Shanghai University of Traditional Chinese Medicine, Shanghai, China; 2 Baoshan Branch, Renji Hospital, School of Medicine, Shanghai Jiao Tong University, Shanghai, China; 3 School of Medicine, Shanghai University, Shanghai, China

**Keywords:** bone and joint pain, exercise therapy, manual therapy, narrative review, rehabilitation

Funding Statement Correction

The funder Advanced Rehabilitation Medical Technology and Management Project of the Shanghai Municipal Health Commission (No. SHKFJS002G) to Jinyan Wang was erroneously omitted.

The complete corrected Funding statement should read: Funding The author(s) declared that financial support was received for this work and/or its publication. This project was supported by the Research Project of Shanghai Second Rehabilitation Hospital (No. Y2025-07), the 2025 Baoshan District Health Commission Outstanding Young Talent (Yucai) Program (No. BSWSYC-2025-17), and the Advanced Rehabilitation Medical Technology and Management Project of the Shanghai Municipal Health Commission (No. SHKFJS002G).

The original version of this article has been updated.

