# Supplementary material for: Advances in rehabilitation for bone and joint pain: a critical narrative review of evidence, gaps, and future directions
Source: Front Pain Res (Lausanne). 2026 Jul 30;7:1858635. doi: 10.3389/fpain.2026.1858635 (PMC13468789; doi:10.3389/fpain.2026.1858635)
Supplement: Supplementary file 1 [file Table1.docx]

# **Supplementary Table 1.** AMSTAR‑2 Assessment for Systematic Reviews

| **AMSTAR‑2 Item** | **Pamboris 2024** | **Lin 2025** | **Jalili Bafrouei 2025** | **Li 2023** | **Luo 2025** | **Xiang 2023** | **Xie 2021** | **Long 2025** | **Nayab 2024** |
| --- | --- | --- | --- | --- | --- | --- | --- | --- | --- |
| **1. PICO components** | Y | Y | Y | Y | Y | Y | Y | Y | Y |
| **2. Protocol registered & justified deviations** | PY | PY | PY | N | PY | N | PY | PY | N |
| **3. Explained study design selection** | Y | Y | Y | Y | Y | Y | Y | Y | PY |
| **4. Comprehensive search strategy** | Y | Y | Y | Y | Y | Y | Y | Y | Y |
| **5. Duplicate study selection** | Y | Y | Y | Y | Y | Y | Y | Y | Y |
| **6. Duplicate data extraction** | Y | Y | Y | Y | Y | Y | Y | Y | Y |
| **7. List of excluded studies with justifications** | N | N | N | N | N | N | N | N | N |
| **8. Adequate description of included studies** | Y | Y | Y | Y | Y | Y | Y | Y | Y |
| **9. Satisfactory RoB assessment technique** | PY | PY | PY | Y | Y | PY | Y | Y | PY |
| **10. Reported funding sources for individual studies** | N | Y | N | Y | N | Y | Y | Y | N |
| **11. Appropriate meta‑analysis methods** | Y | Y | Y | Y | Y | Y | Y | Y | Y |
| **12. Assessed impact of RoB on synthesis** | PY | PY | PY | PY | PY | N | PY | PY | PY |
| **13. Accounted for RoB when interpreting results** | Y | Y | Y | Y | Y | PY | Y | Y | Y |
| **14. Satisfactory explanation & discussion of heterogeneity** | Y | Y | Y | Y | Y | Y | Y | Y | Y |
| **15. Adequate investigation of publication bias** | Y | Y | N | Y | Y | N | N | N | Y |
| **16. Reported potential conflicts of interest** | N | Y | Y | Y | Y | Y | Y | Y | N |

# Y= Yes (fully meets); PY= Partial Yes (meets some sub‑items); N = No

# **Supplementary Table 2.** PEDro Scale Assessment for RCTs

| **Study**  **(Author et al., Year)** | **1. Eligibility criteria** | **2. Random allocation** | **3. Concealed allocation** | **4. Baseline similarity** | **5. Blinding subjects** | **6. Blinding therapists** | **7. Blinding assessors** | **8. >85% follow-up** | **9. Intention- to-treat** | **10. Between- group stats** | **11. Point & variability** | **Total**  **(2-11)** |
| --- | --- | --- | --- | --- | --- | --- | --- | --- | --- | --- | --- | --- |
| **Zhang et al. (2024)** | 1 | 1 | 1 | 1 | 0 | 0 | 1 | 1 | 1 | 1 | 1 | **9** |
| **Massah et al. (2023)** | 1 | 1 | 1 | 1 | 1 | 0 | 1 | 1 | 0 | 1 | 1 | **9** |
| **Werasirirat et al. (2025)** | 1 | 1 | 1 | 1 | 1 | 0 | 1 | 1 | 1 | 1 | 1 | **10** |
| **Erayata et al. (2025)** | 1 | 1 | 1 | 1 | 1 | 0 | 1 | 1 | 1 | 1 | 1 | **10** |
| **Menek et al. (2025)** | 1 | 1 | 1 | 1 | 1 | 0 | 1 | 1 | 1 | 1 | 1 | **10** |
| **Sremakaw et al. (2023)** | 1 | 1 | 1 | 1 | 0 | 0 | 1 | 1 | 1 | 1 | 1 | **9** |
| **Karabay et al. (2025)** | 1 | 1 | 1 | 1 | 1 | 0 | 0 | 1 | 1 | 1 | 1 | **9** |
| **Brelaz et al. (2025)** | 1 | 1 | 1 | 1 | 0 | 0 | 1 | 0 | 1 | 1 | 1 | **8** |
| **Lee et al. (2026)** | 1 | 1 | 1 | 1 | 1 | 0 | 1 | 1 | 1 | 1 | 1 | **10** |
| **Tabassum et al. (2023)** | 1 | 1 | 1 | 1 | 1 | 0 | 1 | 1 | 1 | 1 | 1 | **10** |
| **Pozsgai et al. (2022)** | 1 | 1 | 1 | 1 | 1 | 0 | 0 | 1 | 1 | 1 | 1 | **9** |
| **Ocalan et al. (2025)** | 1 | 1 | 1 | 1 | 0 | 0 | 1 | 1 | 1 | 1 | 1 | **9** |
| **Bağcier et al. (2023)** | 1 | 1 | 1 | 1 | 0 | 0 | 1 | 1 | 1 | 1 | 1 | **9** |
| **Neculăș et al. (2024)** | 1 | 1 | 0 | 1 | 0 | 0 | 1 | 1 | 0 | 1 | 1 | **7** |
| **Zhang et al. (2023)** | 1 | 1 | 1 | 1 | 0 | 0 | 1 | 1 | 1 | 1 | 1 | **9** |
| **Xie et al. (2026)** | 1 | 1 | 0 | 1 | 0 | 0 | 1 | 1 | 1 | 1 | 1 | **8** |
| **Quesnot et al. (2024)** | 1 | 1 | 1 | 0 | 0 | 0 | 1 | 1 | 1 | 1 | 1 | **8** |

**Supplementary Table 3. Full database-specific search strategies**

| **Database** | **Search fields** | **Complete search strategy** | **Limits** | **Records retrieved** |
| --- | --- | --- | --- | --- |
| PubMed (MEDLINE) | Title/Abstract; Publication Type; Date–Publication; Language | (("osteoarthritis"[Title/Abstract] OR "low back pain"[Title/Abstract] OR "patellofemoral pain"[Title/Abstract] OR "shoulder pain"[Title/Abstract] OR "temporomandibular disorders"[Title/Abstract] OR "postoperative pain"[Title/Abstract]) AND (rehabilitation[Title/Abstract] OR "physical therapy"[Title/Abstract] OR "exercise therapy"[Title/Abstract] OR "manual therapy"[Title/Abstract] OR telerehabilitation[Title/Abstract] OR "blood flow restriction"[Title/Abstract] OR "virtual reality"[Title/Abstract]) AND chronic[Title/Abstract] AND (pain[Title] OR musculoskeletal[Title] OR "physical therapy"[Title])) AND (randomized controlled trial[Publication Type] OR systematic review[Publication Type] OR meta-analysis[Publication Type]) AND ("2021/01/01"[Date - Publication] : "2026/04/30"[Date - Publication]) AND English[Language] | English; 1 Jan 2021–30 Apr 2026; RCTs, systematic reviews, and meta-analyses | 365 |
| Embase (Elsevier) | Emtree /exp; title, abstract; publication year; limits | (('osteoarthritis' OR 'low back pain' OR 'patellofemoral pain' OR 'shoulder pain' OR 'temporomandibular disorder*' OR 'postoperative pain'):ti AND (('physical therap*' OR 'exercise therap*' OR 'manual therap*' OR 'blood flow restriction'):ti OR telerehabilitation:ti,ab OR 'virtual reality':ti,ab) AND (chronic OR persistent):ti,ab AND pain:ti) AND [2021-2026]/py AND [english]/lim AND [humans]/lim AND ('randomized controlled trial'/de OR 'systematic review'/de OR 'meta analysis'/de) | English; human studies; 2021–2026; RCTs, systematic reviews, and meta-analyses | 230 |
| Web of Science Core Collection | Topic (TS); document type; language; publication year | TS=((osteoarthritis OR "low back pain" OR "patellofemoral pain" OR "shoulder pain" OR "temporomandibular disorder*" OR "postoperative pain") AND (rehabilitation OR "physical therap*" OR "exercise therap*" OR "manual therap*" OR telerehabilitation OR "blood flow restriction" OR "virtual reality") AND chronic) AND(pain OR musculoskeletal OR "physical therapy"). Refined by: DOCUMENT TYPES=(Article OR Review); LANGUAGES=(English); Publication Years=(2021–2026); Indexes=SCI-EXPANDED and SSCI. | English; article/review; 2021–2026; SCI-EXPANDED and SSCI | 115 |
| PEDro | Advanced search: Title & Abstract; Method; Subdiscipline; Published since; Language | Because PEDro does not support complex nested Boolean syntax in the same manner as PubMed, Embase, or Web of Science, the PEDro Advanced Search was performed using separate Abstract & Title searches and the results were combined before duplicate removal. The following search terms were used in Abstract & Title: "joint pain" rehabilitat*; "joint pain" exercis*; "joint pain" "physical therapy"; "joint pain" "manual therapy"; "bone pain" rehabilitat*; "bone pain" exercis*; "bone pain" "physical therapy"; "bone pain" "manual therapy"; osteoarthrit* rehabilitat*; osteoarthrit* exercis*; osteoarthrit* "physical therapy"; osteoarthrit* "manual therapy"; "chronic pain" rehabilitat*; "chronic pain" exercis*; "chronic pain" "physical therapy"; "chronic pain" "manual therapy". The searches were conducted with Problem = pain, Subdiscipline = musculoskeletal, Published since = 2021, and Language = English. Records were retained when they were clinical trials or systematic reviews relevant to rehabilitation interventions for bone or joint pain. | English; musculoskeletal subdiscipline; clinical trials/systematic reviews; 2021–30 Apr 2026 | 67 |

Selection rationale: records were retained when they provided direct evidence or high-quality contextual synthesis relevant to the review questions. When several records addressed the same question, selection favoured methodological transparency, relevance, and ability to explain agreement, inconsistency, clinical applicability, or evidence gaps. This narrative selection approach was not intended to claim exhaustive identification or statistical synthesis. Database indexing, controlled vocabulary, and interface behaviour change over time; therefore, a later rerun may not return an identical count.
